# Comparison of Hemodynamic Management by Hypotension Prediction Index or Goal-Directed Therapy in Radical Cystectomies: A Prospective Observational Study

**DOI:** 10.3390/jcm14176285

**Published:** 2025-09-05

**Authors:** Claudia Brusasco, Marco Micali, Giada Cucciolini, Desjan Filolli, Michela Gandini, Marco Lattuada, Carlo Introini, Francesco Corradi

**Affiliations:** 1 Anaesthesia and Intensive Care Unit, E.O. Ospedali Galliera, 16128 Genoa, Italy; marco.micali@galliera.it (M.M.); michela.gandini@galliera.it (M.G.); marco.lattuada@galliera.it (M.L.); 2 Department of Surgical, Medical, Molecular Pathology and Critical Care Medicine, University of Pisa, 56124 Pisa, Italy; giadacucciolini@gmail.com (G.C.); desjanfilolli@gmail.com (D.F.); francesco.corradi@unipi.it (F.C.); 3 Departmental Structure of Neuroanesthesia and Intensive Care, Azienda Ospedaliero-Universitaria Pisana, 56124 Pisa, Italy; 4 Department of Abdominal Surgery, Urology Unit, E.O. Ospedali Galliera, 16128 Genoa, Italy; carlo.introini@galliera.it

**Keywords:** intraoperative hypotension, ERAS protocol, invasive and non-invasive hemodynamic monitoring, Hypotension Prediction Index, goal-directed therapy, radical cystectomy, postoperative complications

## Abstract

**Background:** Hypotensive events may occur during surgical interventions and are associated with major postoperative complications, depending on their duration and severity. Intraoperative hemodynamic goal-directed therapy can reduce postoperative complications and mortality in high-risk surgeries and high-risk patients. The study hypothesis was that a proactive approach by hypotension predictive index (HPI) is more effective than a reactive goal-directed therapy (GDT) in reducing the number of hypotensive events during radical cystectomy and that this is associated with improved postoperative outcomes. **Methods:** The study was a single-center prospective observational study conducted at Galliera Hospital, from November 2019 to February 2025, with a before-after population of sixty-seven patients with reactive approach (GDT group) and sixty-five patients with a proactive approach (HPI group) undergoing radical cystectomy, managed with a standardized ERAS protocol and invasive or non-invasive hemodynamic monitoring. The aim of the study was to compare the incidence, duration, and severity of intraoperative hypotensive episodes between a proactive approach guided by the Hypotension Prediction Index (HPI) and a reactive goal-directed therapy (GDT) strategy guided by an advanced hemodynamic monitoring system. **Results:** The HPI group had a 65% reduction in hypotensive events (225 vs. 633, *p* < 0.001), with a 72% reduction in their duration (14 vs. 49 min, *p* < 0.001) and an 85% reduction in their severity (time-weighted average MAP < 65 mmHg 0.11 vs. 0.76, *p* < 0.001) compared to the GDT group. The HPI-guided group showed a reduction in postoperative infectious complications (10 vs. 26) and in-hospital length of stay (8 ± 4 versus 13 ± 8 days). **Conclusions:** A proactive approach may allow attenuating the occurrence and severity of hypotensive events more than a reactive goal-directed approach during radical cystectomy.

## 1. Introduction

Hypotensive events may occur during various types of surgery due to anesthesia and surgical stress. Depending on their severity and duration, these events may be associated with increased risks of perioperative complications, morbidity, and mortality [1,2,3,4,5,6,7,8,9]. Both moderate hypotensive events, defined as mean arterial pressure (MAP) < 65 mmHg lasting >13 min, and severe hypotensive events, defined as MAP < 50 mmHg for ≥1 min, have been reported to increase the risk of myocardial and acute kidney injuries [1,3]. Indeed, goal-directed therapy (GDT) aimed at preventing hypotension by fluid administration has been shown to reduce mortality [10], postoperative acute kidney injury [11], major gastrointestinal complications [12], and postoperative pulmonary complications [13] in high-risk surgeries and high-risk patients. On the other hand, the recently introduced Enhanced Recovery After Surgery (ERAS) protocols [14,15] aim at limiting the overall intravenous fluid infusions to the minimum necessary in order to assure an optimal fluid balance even in the postoperative phase, when oral intake is resumed [16]. Thus, intraoperative advanced hemodynamic monitoring seems to be essential for preventing hypotensive events by optimizing intraoperative fluid administration [10,11,12,13,17].

Radical cystectomy is a complex intervention burdened by frequent complications [18] and hampered by difficult intraoperative fluid management due to unfeasible intraoperative monitoring of urine output. Most perioperative complications, including postoperative acute kidney injury, leakage from ureteral and urethral anastomoses, and paralytic ileus of this surgery, may be linked to inadequate hemodynamic management and fluid balance. Consequently, radical cystectomy performed within the framework of a rigorous ERAS protocol requires fluid management tailored to individual patients throughout the perioperative period [15].

Advanced hemodynamic monitoring and GDT protocols have been developed with hypotension management starting at the onset of the hypotensive event, as detected by the hemodynamic data from the monitoring systems. More recently, a new algorithm named Hypotension Prediction Index (HPI) has been introduced, which may enable a proactive approach with the aim of preventing hypotensive events rather than treating them once they have already occurred. The HPI technology is a machine learning algorithm analyzing changes in arterial waveform distinctive of a compensatory mechanism enacted in response to a transient hypotensive stimulus [17,19,20,21]. A rising HPI value, combined with the continuous analysis of additional macro-hemodynamic indexes, enables the early identification of unstable compensatory mechanisms developing prior to the onset of hypotension [22].

The clinical utility of HPI has been recently challenged by several studies highlighting its technical limitations—such as the use of suboptimal-quality arterial waveforms that frequently occur in operating rooms and make the output unreliable—and questioning the added value of its artificial intelligence (AI) component, which appears to largely reflect mean arterial pressure trends without offering significant incremental predictive value [23,24,25].

Therefore, we designed this prospective observational study to test the hypothesis that a proactive approach based on HPI is more effective than a reactive GDT approach in reducing the incidence and duration of hypotensive events during radical cystectomy.

## 2. Methods

### 2.1. Study Design and Patients

**Figure 1:**
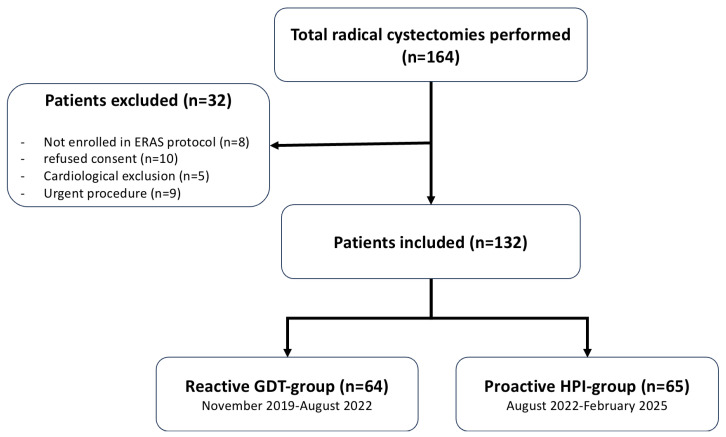
Participant flow diagram.

**Figure 2:**
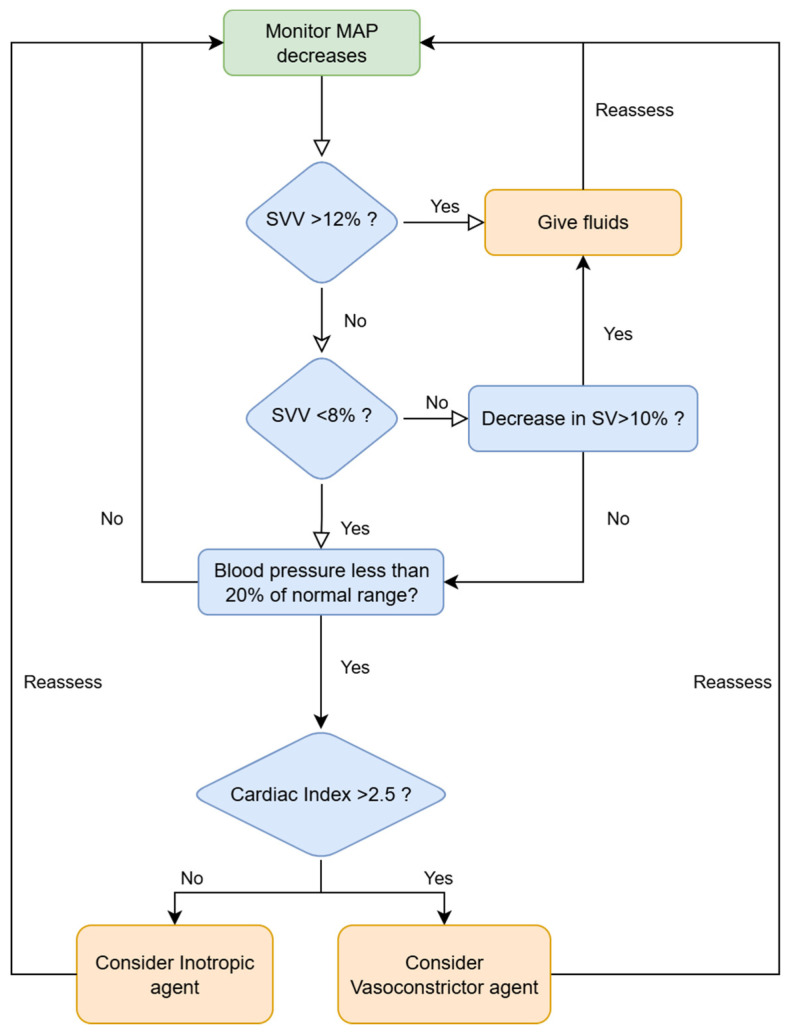
Hemodynamic reactive approach applied to patients monitored through FloTrack or Clearsight sensors (GDT group).

**Figure 3:**
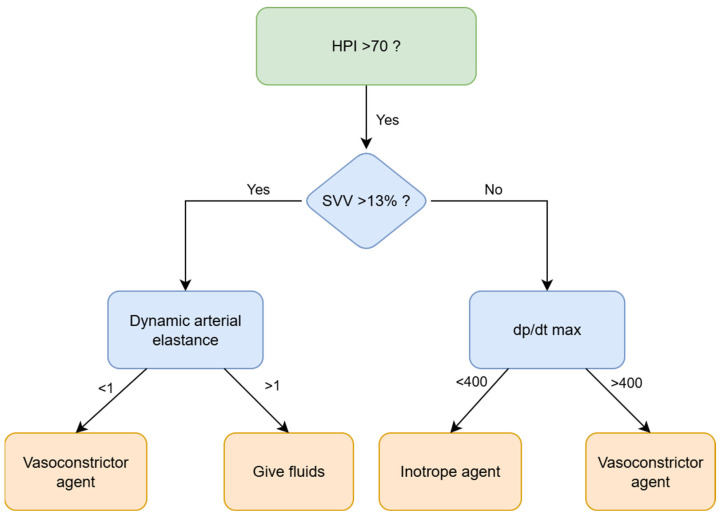
Hemodynamic proactive approach to patients monitored through AcumenIQ or Acumen cuff sensors (HPI group).

This was a single-center, before-after prospective observational study conducted at the Galliera Hospital in Genova from November 2019 to February 2025. All patients signed an informed consent for personal data storage, and the local ethics committee approved the study (7/2019 id: 4378, amendment 2). This manuscript adheres to the applicable STROBE reporting guidelines. The study involved 132 consecutive patients undergoing elective radical cystectomy by open, laparoscopic, or robotic-assisted techniques. Exclusion criteria were: <18 years of age, significant cardiac arrhythmias or aortic regurgitation or cardiac shunt, urgent/emergent surgery, patient’s refusal to treatment of personal data, and patients not enrolled in the ERAS pathway (Figure 1). All patients followed a consolidated perioperative ERAS program as previously described [15] and were accordingly managed following a GDT protocol [26,27,28], guided by an advanced hemodynamic monitoring system (HemoSphere platform, Edwards Lifesciences, Irvine, CA, USA). From November 2019 to August 2022, we used Flowtrack or Clearsight sensors, invasive and non-invasive technology, respectively, adjusting intraoperative fluid administration following stroke volume variation (SVV) or stroke volume (SV) if SVV was not reliable due to atrial fibrillation, cardiac index (CI), and mean arterial pressure (MAP) (Figure 2—reactive GDT group) [29]. Since September 2022, we have upgraded our monitoring technology by introducing the Acumen IQ and Acumen cuff, invasive and non-invasive technology, respectively, a proactive system capable of providing additional parameters, such as Hypotension Prediction Index (HPI), arterial dynamic elastance (Eadyn), and left and right ventricular contractility (dP/dt). (Figure 3—HPI group) [21,30]. HPI is a unitless number, ranging from 0 to 100, which gives the likelihood that a hypotensive event with a MAP < 65 mmHg lasting for >1 min will occur within 5–15 min, even if the patient is still normotensive. The higher the HPI value, the higher the risk of a hypotensive event [20,31]. An HPI value > 85 was originally proposed as a threshold indicating a very high likelihood of impending hypotension. However, in our clinical practice, in accordance with our current operative protocol, we have opted to base hemodynamic monitoring on the observation of trends. Particular attention is paid to upward trajectories, and clinical concern arises when HPI increases by more than 20% from baseline. In such cases, we elect to intervene at a lower threshold of HPI > 70. Upon reaching this threshold, the subsequent step consistently involves a time-based analysis of the extent and duration of variations in the three macro-hemodynamic parameters available to us: stroke volume variation (SVV), the rate of pressure change (dP) with time (dt) during isovolemic contraction of the cardiac ventricles (dP/dt), and dynamic arterial elastance (Eadyn). This approach has enabled a more nuanced assessment of whether the evolving hemodynamic disturbance is primarily attributable to alterations in preload, myocardial contractility, or afterload.

Patients were monitored using non-invasive sensors (Clearsight or Acumen Cuff) whenever feasible as the first-line strategy. In cases of predicted high risk of intraoperative hemorrhage (advanced oncologic status, nerve-sparing surgery, or previous neoadjuvant chemotherapy) or a known history of severe vasculopathy, invasive monitoring (FloTrac or Acumen IQ) was employed.

### 2.2. ERAS Protocol and Intraoperative Procedures

All patients avoided fasting and thirst by eating until six hours before anesthesia and drinking oral carbohydrates the night before and two hours before surgery. Once in the operating room, a thoracic epidural catheter was placed. Patients were then managed following an intraoperative GDT guided by hemodynamic monitoring as per our ERAS protocol [15], positioning either the invasive sensor through a radial artery or the non-invasive sensor through the finger cuff before anesthesia induction depending on the clinician’s decision. General anesthesia was maintained in a blended manner with a sevoflurane minimum alveolar concentration of 0.5–0.6%, and lidocaine infusion (2 mg/kg/h) kept the bi-spectral index value between 60 and 65, and boluses of ropivacaine 0.375–0.250% in the peridural catheter were repeated every 90 min. Deep neuromuscular blockage was maintained throughout the surgery with a train-of-four of 0 and a post-tetanic count < 4. Pressure-controlled ventilation with guaranteed volume was used for intraoperative mechanical ventilation. Tidal volume was set at 7–8 mL/kg, and respiratory rate was adjusted to maintain an end-tidal CO_2_ of 30–35 mmHg. For minimally invasive radical cystectomies, either laparoscopic or robot-assisted, a pneumoperitoneum pressure of 10 mmHg was applied by using the AirSeal Intelligent Flow System^®^ (ConMed, Utica, NY, USA) or the Lexion System^®^ (Lexion Medical, St. Paul, Minnesota, USA) throughout the surgery in a Trendelenburg position ranging between 20 and 24°. For open surgery a Trendelenburg position of 5–8° was maintained. After surgery and extubation, patients were monitored for 60 min in the recovery room and discharged when the modified Aldrete score was >8. Postoperatively, all patients received crystalloid infusion at 1 mL/kg/h for 18 h. Two hours after surgery, patients started drinking water; on the morning of postoperative day 1, i.v. fluid administration was discontinued, and free oral water intake allowed.

### 2.3. Data Collection

After multidisciplinary counseling in the context of preoperative evaluation, patients were enrolled in the ERAS protocol, and all demographic, anamnestic, and clinical data were stored. Intraoperatively, the anesthesiologist manually recorded the type of anesthesia, surgery duration, intraoperative blood loss, use of norepinephrine infusion, and total amount of fluids administered. The hemodynamic data recorded by the HemoSphere advanced monitoring platform every 20 s were digitally extrapolated and analyzed by specific software (Acumen Analytics software, version 2.0.0, Edwards Lifesciences, Irvine, CA, USA), providing for each patient the number of total (MAP < 65 mmHg) or severe (MAP < 50 mmHg) hypotensive events, their mean duration, and time-weighted average (TWA), which is the area under the MAP values divided by the total duration of surgery. Postoperative data and outcomes were recorded according to our ERAS protocol, i.e., onset of postoperative acute kidney or myocardial injuries, major complications before hospital discharge, days of hospitalization, and readmission at 30 days.

### 2.4. Aim of the Study

The aim of our study was to compare incidence, duration, and severity of intraoperative hypotensive events between the reactive and the proactive groups. The hypotensive events were classified as moderate (MAP < 65 mmHg) or severe (MAP < 50 mmHg) for at least 1 min.

### 2.5. Statistical Analyses

Normality was tested by Shapiro–Wilk’s test. Comparisons between groups were performed using Student’s *t*-test or the Mann–Whitney U-test for continuous variables and the χ^2^ test for categorical variables. Median differences and respective 95% confidence intervals were calculated with the Hodges–Lehmann method. Continuous data are reported as mean ± standard deviation (SD) or as median [interquartile range]; categorical data are reported as numbers (percentages). A *p*-value of ≤0.05 was considered statistically significant. The SPSS v23.0 software (SPSS Inc., Chicago, IL, USA) was used for all analyses.

## 3. Results

There were no significant differences between the proactive (HPI) and the reactive (GDT) groups. in terms of age, sex, BMI, ASA physical status, comorbidities, preoperative blood tests, type of urinary diversion, or duration of surgery (Table 1). Similarly, no statistically significant differences were observed in the number of patients requiring intraoperative norepinephrine infusion or in intraoperative blood loss. However, the volume of intraoperative fluid administration was significantly lower in the proactive HPI group compared to the reactive GDT group (*p* = 0.036) (Table 2).

**Figure 4:**
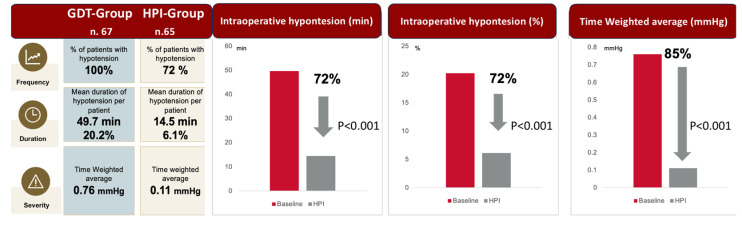
Frequency, duration, and severity of hypotensive events in the reactive GDT group and in the proactive HPI group.

In the HPI group, hypotensive events were 65% less frequent (225 vs. 633, *p* < 0.001), 72% shorter in duration (14 vs. 49 min, *p* < 0.001), and 85% less severe, as evidenced by a significantly lower time-weighted average (0.11 vs. 0.76, *p* < 0.001), compared to the GDT group (Figure 4).

Regarding severe hypotensive episodes, the number of patients experiencing a MAP < 50 mmHg was 73% lower in the HPI group than in the GDT group (9 vs. 33, *p* < 0.001). Additionally, these episodes were 67% less severe, as documented by the time-weighted average (0.02 vs. 0.06, *p* < 0.001), and 72% shorter in duration (0.8 vs. 2.8 min, *p* < 0.001).

**Figure 5:**
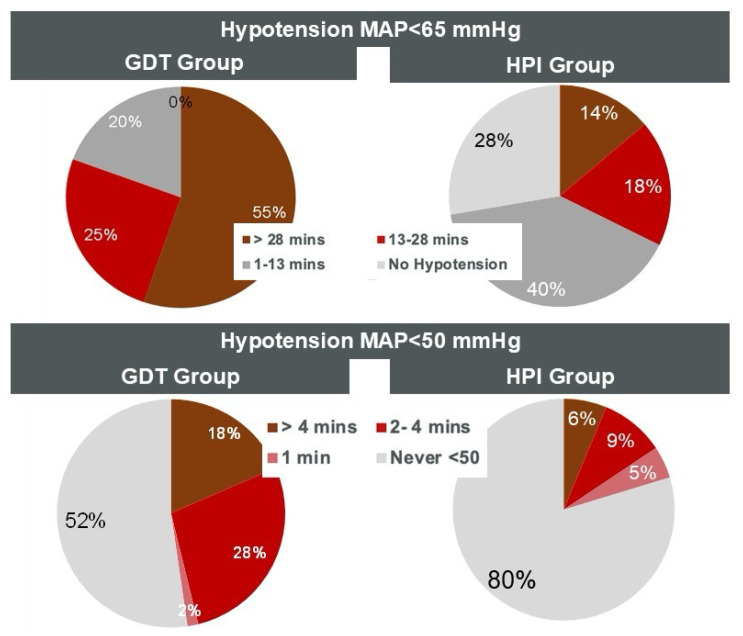
Distribution of duration of hypotensive events for MAP < 65 mmHg and MAP < 50 mmHg in the reactive GDT group and in the proactive HPI group.

In the GDT group, 80% of patients experienced at least one hypotensive event with MAP < 65 mmHg lasting more than 13 min, compared to only 32% in the HPI group. Furthermore, a severe hypotensive episode (MAP < 50 mmHg lasting more than 1 min) occurred in 46% of GDT patients but only 15% of HPI patients (Figure 5).

The HPI group also showed fewer postoperative infectious complications and shorter in-hospital length of stay than the GDT group (Table 2).

**Figure 6:**
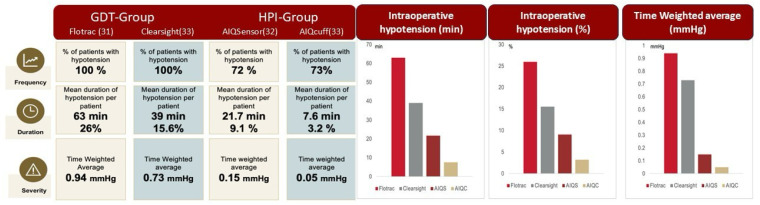
Comparison of frequency, duration, and severity of hypotensive events in the reactive GDT group and in the proactive HPI group divided per non-invasive or invasive monitoring tool.

The number of patients managed with invasive vs. non-invasive hemodynamic monitoring sensors was 31 vs. 33 in the GDT group and 32 vs. 33 in the HPI group, respectively (Figure 6). In all patients monitored non-invasively, continuous and reliable hemodynamic data were successfully maintained throughout the entire surgical procedure, with no interruptions or need for conversion to invasive monitoring.

## 4. Discussion

The main findings of this observational study were that a proactive approach guided by HPI for hemodynamic management (1) was superior to the reactive GDT approach in decreasing incidence, duration, and severity of intraoperative hypotensive events in patients undergoing a radical cystectomy following an ERAS protocol and (2) a proactive approach further reduced intraoperative fluid infusions, postoperative infectious complications, and hospitalization time.

Our results on the reduction of the severity of intraoperative hypotensive events are in line with several previous single-center trials [22,32,33,34,35]. In previous studies, the time-weighted average for MAP < 65 mmHg was reduced by HPI guidance from 0.44 to 0.10 (77%) [17], from 0.50 to 0.16 mmHg (68%) [20], and from 0.27 to 0.10 mmHg (63%) [32]. Tsoumpa et al. reported a 68% reduction in the time-weighted average for MAP < 65 mmHg using the HPI sensor, but also an increase in hypertension and phenylephrine use, identifying an increased risk of over-treatment [20]. In our HPI group, the time-weighted average for MAP < 65 mmHg was 0.11 mmHg, with an 85% reduction compared to 0.76 mmHg in the GDT group, without increasing the use of vasoconstrictors.

These differences may be explained by our intraoperative hemodynamic monitoring strategy, which did not rely primarily on the absolute numerical value of the HPI or the AI component of the software. Instead, we deliberately prioritized human physiological evaluation over algorithmic intelligence, thus guiding anesthetic management according to real-time physiological trends throughout surgery in response to surgical stimuli and administered drugs, without placing undue confidence in raw numerical outputs. We did so based on the belief that no single numerical value, however sophisticated, can adequately capture the complexity of physiology and pathophysiology in each individual patient undergoing major surgery. In this context, where multiple unforeseen events may occur and alter the clinical trajectory, no AI can reliably predict events that are, by definition, unexpected or absent from its training data—particularly when such data are of suboptimal or distorted quality [36]. By contrast, physiological trends remain meaningful, even when macro-hemodynamic variables appear stable. Given the marked inter-individual variability in response to anesthetic agents and vasoactive drugs—partly determined by the patient’s baseline cardiovascular compliance—our protocol incorporates a baseline hemodynamic assessment, followed by an early evaluation after both anesthesia induction and pneumoperitoneum establishment. Then, intraoperative management is guided by the analysis of trend variations, with particular attention to significant changes defined as HPI increases of >20% from baseline.

We choose to act at a lower HPI threshold of 70—rather than the originally proposed 85—to ensure timely responses. In our experience, planning interventions at HPI ≥ 85 often leaves insufficient time to implement appropriate anesthetic strategies to avoid hypotension. By lowering the intervention threshold, we were able to anticipate hemodynamic deterioration and initiate minimal corrective actions. This strategy likely contributed to the observed reduction in intraoperative hypotensive events, without increasing fluid administration or causing overshooting with vasopressors. It enabled timely, physiologically grounded interventions targeting the underlying mechanism of hemodynamic imbalance—whether preload, contractility, or afterload—guided by trend analysis rather than absolute values.

Corrective actions—vasopressors, fluids, or inotropes—were tailored according to the interpretation of variations in three machine learning-derived indices: SVV, dP/dt, and EaDyn. These indices support inference on the predominant cause of hypotension, distinguishing between vasodilation (e.g., due to anesthetic agents), hypovolemia, and reduced cardiac contractility [37].

Notably, recent prospective research demonstrated that a MAP threshold of 72–73 mmHg corresponds to the HPI > 85 alert in 97% of cases and provides equivalent predictive performance for intraoperative hypotension [24,38]. This observation may explain conflicting results in the literature regarding HPI use, suggesting that the benefits observed in our cohort may stem not solely from the proprietary algorithm performance but from its capacity to enhance the anesthetist’s vigilance and promote time-based interpretation of macro-hemodynamic changes.

Our study aimed to compare a proactive HPI-guided strategy with a reactive approach still based on standard hemodynamic monitoring. Therefore, we cannot conclude that the benefits observed are attributable to the HPI algorithm per se, but rather to its application within our specific clinical protocol. Theoretically, similar outcomes might have been achieved using continuous MAP monitoring with an appropriately selected preemptive intervention threshold.

Nonetheless, we believe it remains valuable to have a secondary display—such as that provided by HPI—that enables dynamic trend analysis of contractility and aortic elastance, assisting clinicians in hypothesizing the predominant mechanism underlying hypotensive events.

Educational strategies focused on raising awareness of intraoperative hypotension risks and promoting physiologically driven, individualized interventions are highly desirable and may suffice for managing most surgical patients [39]. More advanced tools such as HPI may be best reserved for highest-risk cases. Future studies comparing proactive strategies based on either HPI or MAP trends could help to clarify the true clinical value of the HPI algorithm.

However, this approach enabled us not only to be more precise but also to move beyond the outdated concept of fluid responsiveness. Our focus was on optimizing the major macro-hemodynamic determinants of organ perfusion while minimizing the need for fluid boluses. Indeed, it is now evident that being a fluid responder is an independent concept and does not necessarily indicate the need for fluids, or vice versa. Thus, our goal shifted toward maintaining tissue perfusion while avoiding organ congestion due to fluid overload. Moreover, the intraoperative strategy, when vasopressor support was required, consisted of initiating an early low-dose norepinephrine infusion rather than administering repeated phenylephrine boluses. This approach likely provided more stable hemodynamic control by avoiding the pressure surges associated with bolus dosing.

ERAS protocols are particularly focused on fluid management from the preoperative to postoperative period. In a previous study [15], we demonstrated a significant reduction in intraoperative fluid administration in radical cystectomies performed after ERAS protocol implementation and managed with a GDT protocol, as compared to those performed before ERAS protocol implementation and managed without advanced hemodynamic monitoring systems.

Several previous studies, either observational or randomized, could not find significant differences in clinical outcomes despite a significant reduction in the number, duration, and severity of intraoperative hypotensive events [20,33,35,40,41]. Few retrospective clinical studies found reductions in hypotension, postoperative complications, and length of stay with HPI guidance, but none found significant reductions in acute kidney injury [42,43]. Recent systematic meta-analyses confirmed the robust predictive accuracy of HPI (AUC ≈ 0.9) and its ability to reduce the burden of intraoperative hypotension when integrated into treatment protocols [38,44]. However, they did not show evidence in postoperative outcome improvements, probably mostly due to a significant heterogeneity between studies, intervention thresholds, and clinical endpoints [45]. These inconsistencies of outcomes may be due to two main factors. First, the few randomized trials were not powered to detect effects on all postoperative outcomes, while observational or retrospective studies were marred by several confounding factors, making it impossible to establish strict relationships between hypotension and single adverse events. Second, several studies on non-cardiac surgery lacked uniformity in both perioperative management and surgical procedures [41]. We studied a homogeneous population made of patients undergoing radical cystectomy and following the same standardized strict ERAS protocol certified by the ERAS^®^ Society. This might represent a major strength of our study. The proactive approach following HPI monitoring, associated with a significant reduction in intraoperative hypotensive events, appears to have a positive impact particularly on the incidence of postoperative infectious complications and overall length of hospital stay.

A potential mechanism for the reduction in postoperative infections could have been an improved splanchnic perfusion, which is recognized as a key trigger for bacterial translocation, sepsis, and ultimately multi-organ failure [46,47,48]. On the other end, limiting fluid boluses and avoiding fluid overload may prevent AKI and splanchnic congestion, which may be both associated with increased postoperative infections [49]. However, in the absence of microcirculation data and lactate measurements, these hypotheses are speculative and require further investigation by randomized controlled trials powered for clinical outcomes and splanchnic perfusion measures.

In the current study, we used either invasive or non-invasive sensors in both groups, with the choice depending on anesthesiologists’ clinical decisions mainly based on estimated hemorrhagic risk and the patient’s peripheral perfusion. Non-invasive monitoring was effective, reliable, and easy-to-use.

This study has several limitations. First, because of its prospective observational nature, the results need to be confirmed by further randomized controlled studies. Moreover, due to the five-year length of study collecting consecutive patients treated in two different ways in a before-after manner, a temporal bias might be taken into account. The extremely consistent ERAS program developed since 2018 for all radical cystectomies might have partially guaranteed no changes in clinical practice, staff awareness, or other factors influencing outcomes, thus reducing the risk of important temporal biases. However, the prospective observational nature of the study might also be a strength of it, because in previous randomized trials physicians were using one or the other hemodynamic system, being aware of the existence of HPI and the proactive approach with a consequent risk of bias related to possible over-treatment of control cases, while in our study the entire GDT group was collected before the development and marketing of the hypotension predictive index, thus the anesthesiologist was completely unaware of the proactive approach. Second, the small sample size of this observational study was underpowered for rare outcomes. Third, myocardial injury might have been overlooked due to lack of routine postoperative measurements of troponin concentration. Fourth, being all patients undergoing radical cystectomies, postoperative acute kidney injury might have been partly due to obstructive causes such as ureteral edema or clot formation. Fifth, lactates were not collected at the end of surgery and postoperatively, thus making any comparisons of effect on tissue perfusion impossible. Sixth, this study encompassed all surgical approaches—open, video laparoscopic, and robotic. It should be acknowledged that the accuracy and validity of the monitored hemodynamic parameters may be influenced by intraoperative factors specific to minimally invasive techniques, such as the application of pneumoperitoneum and the use of Trendelenburg positioning. No stratified analysis was performed to compare monitoring outcomes across different surgical approaches, which may have limited the ability to detect approach-specific variations. Nevertheless, the use of parameter trends referenced to each patient’s baseline physiology allowed meaningful interpretation of the monitoring data, even in the context of potentially reduced validity of absolute parameter values.

## 5. Conclusions

The results of this study suggest that, in patients undergoing radical cystectomy following a strictly standardized ERAS protocol, the use of a proactive HPI-guided approach effectively reduces the frequency, duration, and severity of intraoperative hypotensive events. This hemodynamic optimization was achieved with a significant reduction in fluid administration and without an increase in vasoconstrictor use. However, further large-scale, multi-center randomized controlled trials are required to comprehensively assess postoperative outcomes, avoiding the possible effect of increased staff vigilance and expertise and surgical advancement. The next crucial gap to address is the development of monitoring systems capable of directly assessing splanchnic perfusion. This would allow clinicians to titrate macro-hemodynamic parameters according to actual organ perfusion, enabling truly individualized treatment strategies aimed at determining the optimal mean arterial pressure for each patient.

## Figures and Tables

**Table 1:** Demographic data and baseline characteristics.

	GDT Group (n.67)	HPI Group (n.65)	*p*-Value
Age (yr)	74 [66–78]	74 [67–79]	0.742
Sex, m/f (n)	56/11	55/10	0.910
BMI (kg/m^2^)	25 [23–28]	26 [23–29]	0.158
ASA physical status			
I	0	0	
II	39	35	
III	29	29	0.563
IV	0	1	
Previous medical history (n)			
Chronic congestive heart failure	3	1	0.62
Coronary disease	7	4	0.532
Atrial fibrillation and/or valvopathy	7	6	0.535
Smoking	27	19	0.274
COPD	13	7	0.227
Diabetes	12	10	0.817
Previous cerebrovascular event	3	5	0.486
Chronic AKI	21	13	0.168
Preoperative hemoglobin (g/dL)	13.2 [11.8–14.3]	12.8 [11.3–14.3]	0.317
Preoperative creatinine (mg/dL)	1.1 [0.88–1.3]	1.1 [0.9–1.4]	0.636
Preoperative eGFR (mL/min/1.73 m^2^)	64.1 [46.9–74.9]	60.1 [45.1–80.1]	0.357
Video laparoscopic or robotic surgery (n)	25	37	0.06
Type of surgery (n)			
Open surgery	42	28	
Video laparoscopic	25	17	
Robotic surgery	0	20	
Duration of surgery (min)	205 [180–240]	240 [195–318]	0.07
Type of urinary diversion (n)			
Orthotopic	13	5	
Bricker	31	27	
Ureterocutaneostomy	23	33	

BMI, Body Mass Index; ASA, American Society of Anesthesiology; COPD, chronic obstructive pulmonary disease; AKI, acute kidney injury; eGFR, glomerular filtration rate; GDT, goal-directed therapy; HPI, hypotension predictive index.

**Table 2:** Hemodynamic and clinical data and outcomes.

	GDT Group (n.67)	HPI Group (n.65)	*p*-Value
Patients requiring noradrenaline (n)	14	10	0.503
Crystalloids infused during surgery (mL)	2750 [1700–2500]	1700 [1450–2200]	**0.036**
Blood loss during surgery (mL)	400 [213–600]	400 [200–750]	0.594
Total hypotensive events (n)	633	225	**<0.001**
Time-weighted average of MAP < 65 mmHg	0.76 [0.4–1.75]	0.11 [0.01–0.34]	**<0.001**
Patients with hypotensive events (n)	67	47	**0.03**
Hypotensive events < 65 mmHg duration (min)	3 [2–6]	2 [1–4]	**<0.001**
Total time in hypotension < 65 mmHg (min)	32 [14.8–64.7]	3.3 [0–21]	**<0.001**
Hypotension < 65 mmHg (% of surgery time)	14.1 [6.8–30.1]	1.6 [0–7.9]	**<0.001**
Time-weighted average for MAP < 50 mmHg	0.06 [0–0.14]	0.02 [0–0.6]	**<0.001**
Patients with hypotensive events < 50 mmHg (n)	33	9	**<0.001**
Hypotension < 50 mmHg (% of surgery time)	1.2 [0.1–3.1]	0.3 [0.1–0.84]	**<0.001**
Hemoglobin at discharge (g/dL)	10.3 [9.4–11.3]	8.3 [9.6–11.8]	0.142
Creatinine at discharge (mg/dL)	1.1 [0.8–1.3]	1.0 [0.8–1.45]	0.614
eGFR at discharge (mL/min/1.73 m^2^)	64.9 [45.5–84.1]	64.6 [44.1–93.3]	0.267
Intensive care unit admittance (n)	12	4	0.05
Total major postoperative complications (n)	28	23	0.593
Postoperative acute kidney injury (n)	20	18	0.512
Kidgo 1	12	8	0.472
Kidgo 2	3	4	0.712
Kidgo 3	5	6	0.759
Postoperative cardiovascular complications (n)	0	0	0.999
Infectious postoperative complications (n)	26	10	**0.003**
Reintervention (n)	6	6	0.999
Anastomotic dehiscence or urinary leakage (n)	5	6	0.76
In-hospital length of stay (days)	10 [8–15]	7 [6–9]	**<0.001**
Readmission at 30 days from surgery (n)	16	13	0.66

## Data Availability

Data was not inserted in publicly archived datasets but is available anonymized for research purposes if asked to corresponding author.
